# Galectin-3 modulates epithelial cell adaptation to stress at the ER-mitochondria interface

**DOI:** 10.1038/s41419-020-2556-3

**Published:** 2020-05-12

**Authors:** Lucie Coppin, Arnaud Jannin, Emilie Ait Yahya, Caroline Thuillier, Céline Villenet, Meryem Tardivel, Antonino Bongiovanni, Cécile Gaston, Simon de Beco, Nicolas Barois, Isabelle van Seuningen, Emmanuelle Durand, Amélie Bonnefond, Jean-Claude Vienne, Joseph Vamecq, Martin Figeac, Audrey Vincent, Delphine Delacour, Nicole Porchet, Pascal Pigny

**Affiliations:** 1 University of Lille, CNRS, Inserm, CHU Lille, UMR9020 – UMR-S 1277 - Canther – Cancer Heterogeneity, Plasticity and Resistance to Therapies, F-59000 Lille, France; 2 CHU Lille, Institut de Biochimie & Biologie Moléculaire, Centre de Biologie et Pathologie, F-59000 Lille, France; 3 CHU Lille, Institut de Génétique, Centre de Biologie et Pathologie, F-59000 Lille, France; 4 University of Lille, CHU Lille, Plate-forme de génomique fonctionnelle, Centre de Biologie et Pathologie, F-59000 Lille, France; 5 University of Lille, F-59000 Lille, France; 6 Equipe Cell adhesion and mechanics, Institut Jacques Monod - UMR 7592 CNRS - Université Paris Diderot, F-75205 Paris Cedex 13, France; 7 University of Lille, CNRS, Inserm, CHU Lille, Institut Pasteur de Lille, U1019 - UMR 8204 - CIIL - Center for Infection and Immunity of Lille, F-59000 Lille, France; 8 University of Lille, CNRS, CHU Lille, Institut Pasteur de Lille, UMR 8199, EGID, F-59000 Lille, France

**Keywords:** Cancer metabolism, Endoplasmic reticulum, Energy metabolism

## Abstract

Cellular stress response contributes to epithelial defense in adaptation to environment changes. Galectins play a pivotal role in the regulation of this response in malignant cells. However, precise underlying mechanisms are largely unknown. Here we demonstrate that Galectin-3, a pro and anti-apoptotic lectin, is required for setting up a correct cellular response to stress by orchestrating several effects. First, Galectin-3 constitutes a key post-transcriptional regulator of stress-related mRNA regulons coordinating the cell metabolism, the mTORC1 complex or the unfolded protein response (UPR). Moreover, we demonstrated the presence of Galectin-3 with mitochondria-associated membranes (MAM), and its interaction with proteins located at the ER or mitochondrial membranes. There Galectin-3 prevents the activation and recruitment at the mitochondria of the regulator of mitochondria fission DRP-1. Accordingly, loss of Galectin-3 impairs mitochondrial morphology, with more fragmented and round mitochondria, and dynamics both in normal and cancer epithelial cells in basal conditions. Importantly, Galectin-3 deficient cells also display changes of the activity of the mitochondrial respiratory chain complexes, of the mTORC1/S6RP/4EBP1 translation pathway and reactive oxygen species levels. Regarding the ER, Galectin-3 did not modify the activities of the 3 branches of the UPR in basal conditions. However, Galectin-3 favours an adaptative UPR following ER stress induction by Thapsigargin treatment. Altogether, at the ER-mitochondria interface, Galectin-3 coordinates the functioning of the ER and mitochondria, preserves the integrity of mitochondrial network and modulates the ER stress response.

## Introduction

Eukaryotic cells are partitioned into discrete organelles endowed with specific functions. For example, protein synthesis, folding, secretion and degradation take place at the endoplasmic reticulum (ER), whereas cellular bioenergetics occurs at the mitochondria. Cell homeostasis depends on the ability of these distinct organelles to communicate and to exchange cargos. Among them, the ER appears especially important since it can develop close contacts with most organelles, notably the mitochondria^1^. Interestingly, the ER-mitochondria interactions which are commonly referred to as mitochondria-associated membranes (MAM) regulate important cellular processes such as mitochondrial biogenesis and metabolism, calcium signalling or lipid biosynthesis^2,3^. When the protein synthetic load overcomes the ER folding capacities, a state called ER stress occurs. To restore ER homeostasis, a network of transduction pathways named unfolded protein response (UPR) is activated^4^. Collectively, these pathways act to reduce protein synthesis and to increase ER folding and ER-associated degradation (ERAD) capacities. However, when this adaptative response fails the UPR evolves towards apoptosis.

Galectin-3 is a β-galactoside-binding lectin, encoded by *LGALS3* gene in humans, which contains a C-terminal carbohydrate recognition domain (CRD) responsible for interactions with glycolipids or glycoproteins and a low complexity domain which allows interactions with the CRD and other partners^5,6^. Moreover, despite the absence of a canonical RNA-binding domain, Galectin-3 is a non-classic RNA-binding protein (RBP) able to stabilise mucin *MUC4* mRNAs in cancer cells^7^. Galectin-3 is highly expressed by epithelial cells and plays important roles in the organisation of renal and intestinal cells. Although Galectin-3-KO mice are viable in controlled conditions, loss of Galectin-3 leads to morphological abnormalities of the epithelial cells as well as perturbation of the biosynthetic pathway^8–10^. Galectin-3 is a soluble protein which is synthesised on free ribosomes and thus bypasses the classical ER-Golgi pathway for its secretion in the extracellular medium. Indeed, premature binding of Galectin-3 with its ligands which are major components of the ER lumen would cause aggregation and perturb the secretory pathway^11,12^. While being synthesised in the cytosol, Galectin-3 associates with various organelles, such as carrier vesicles or endosomes^13^. At the mitochondria level, Galectin-3 prevents the cytochrome-c release and ensures mitochondrial integrity^14,15^. However, it is currently unknown whether these mitochondrial effects depend on Galectin-3 ability to modulate mitochondria-ER interactions in epithelial cells.

In the present study we first aimed to obtain a global view of the post-transcriptional regulatory action of Galectin-3 in epithelial cells. To this end, we combined whole transcriptome stability analysis with mRNA and protein quantification. We showed that Galectin-3 regulates the stability of subsets of mRNAs which share similar functions notably cell metabolism, cell death and stress response pathways. By coupling imaging and biochemical approaches, we showed that Galectin-3 localises at the ER-mitochondria interface where it preserves the integrity of the mitochondrial network and modulates the cellular bioenergetics and the UPR.

## Results

### Gal-3 regulates the half-life of subsets of mRNAs with shared functions

**Fig. 1:**
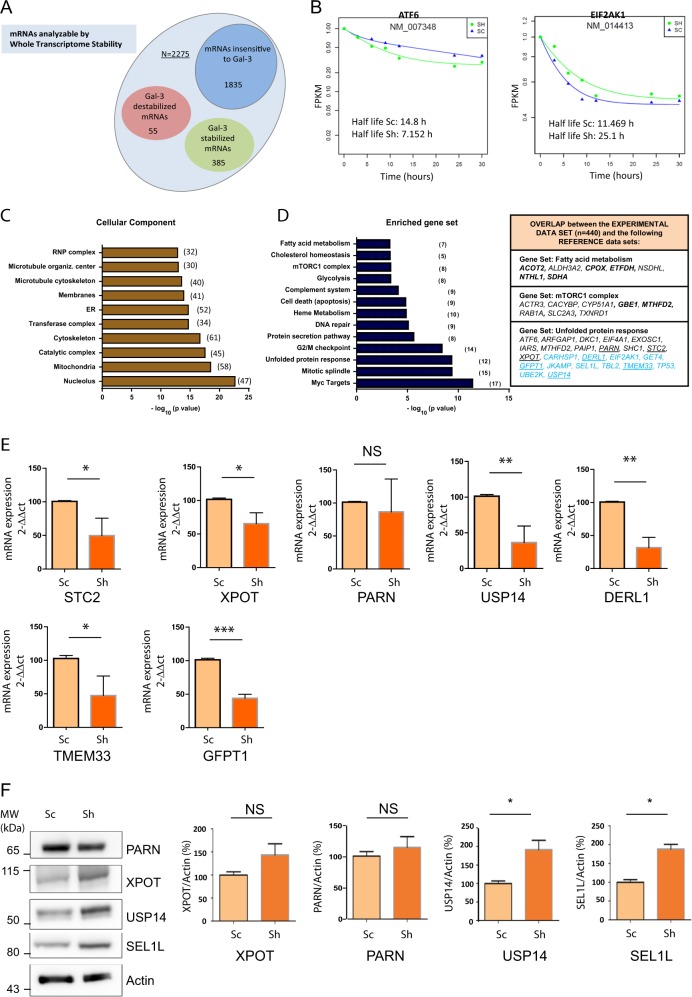
Galectin-3 regulates the half-life of more than 400 mRNAs which shared similar functions. **a** Total number of mRNA studied by whole transcriptome stability analysis in pancreatic Sh and control Sc cells (see text for details). **b** Example of one Galectin-3 stabilised (*ATF6*) and one Galectin-3 destabilised mRNA (*EIF2AK1*). **c**, **d** Significantly enriched organelles (**c**) or biological pathways (**d**) to which belong the mRNA stabilised or destabilised by Galectin-3 (*n* = 440) as determined by enrichment analysis performed with the GSEA tool (the number of mRNAs in the experimental dataset which overlaps with the reference datasets is shown under brackets; horizontal bar indicates the p value of the Fisher’s exact test (−log10). Examples of mRNA species stabilised/destabilised by Galectin-3 belonging to three different pathways are shown in the table (mRNA species in black were assigned to the biological category by the GSEA analysis whereas those in blue were manually assigned based on AmiGO-derived annotation; in bold dark are shown mRNAs which were assigned to mitochondria as cellular component by GSEA; underlined are shown mRNAs which were studied in **e**). **e** Expression levels of selected mRNA species stabilised by Galectin-3 and belonging to the UPR pathway were determined by qRT-PCR in Sh and control Sc cells at baseline (*n* = 3 independent experiments). **f** Expression levels of four selected proteins and actin determined by western blotting (*n* = 3 independent experiments). Fold change was analysed by densitometry. Expression level was arbitrarily set at 100% for Sc cells. Data are mean ± SEM; *p < 0.05, **p < 0.01, ***p < 0.001 and NS not significant.

We first aimed to obtain a global view of the action of Galectin-3 as a post-transcriptional regulator in epithelial cells. For that purpose, we used two models deriving from the human pancreatic cancer cell line T3M-4, control Sc cells expressing high levels of Galectin-3 and a representative mutant clone (Sh1 called Sh cells thereafter) where Galectin-3 expression was stably knocked-down by *sh*RNA (Fig. S1). Using actinomycin D and RNA-Seq, we performed a whole-transcriptome stability analysis. We built the decay curve for 9444 transcripts in both models using the most appropriate kinetic model. In all, 3356 transcripts with a decay curve characterised by a *r*^2^ ≥ 0.9 were selected for further analysis. Rapidly decaying mRNAs (*t*_1/2_ < 3.0 h) were excluded as the experimental time-points did not allow reliable calculation of their half-lives. Therefore, the total number of RNAs analysable was 2275 (Fig. 1a). A total of 440 mRNAs showed a ≥2-fold difference in their half-life between Sc and Sh cells (Table S1). The vast majority (385 mRNAs) were stabilised by Galectin-3 whereas only 55 mRNAs were destabilised by Galectin-3 (Fig. 1a, b and Table S1). To further identify the biological processes and the organelles involved, the set of mRNAs whose stability was modified by Galectin-3 (*n* = 440) was submitted to an analysis by the Gene Set Enrichment Analysis (GSEA) tool (Fig. 1c, d). Focusing on cellular component, the mitochondria and the ER were significantly enriched in proteins whose mRNAs are post-transcriptionally regulated by Galectin-3 (Fig. 1c, Table S2). Using Hallmark as a reference collection, 13 biological pathways were significantly enriched in our data set, several previously unrelated to Galectin-3 such as metabolic pathways (i.e. glycolysis, fatty acid metabolism) and the UPR (Fig. 1d and Table S2). Regarding UPR, twelve mRNAs stabilised by Gal-3 were classified in this category by GSEA (in black). Twelve additional mRNAs (in blue) were assigned to UPR based on the Gene Ontology (GO) annotation of the gene product as determined with AmiGO (Fig. 1d). To check whether changes of the mRNA half-life influence mRNA levels, we evaluated by qPCR the steady state mRNA levels of 7 mRNA from the UPR category (Fig. 1d, underlined) and stabilised by Galectin-3. All but one mRNA showed a significant reduction of expression in Sh cells by comparison with Sc cells (Fig. 1e). Moreover, we studied protein expression of four mRNA stabilised by Galectin-3, namely PARN, XPOT, USP14 and SEL1L in basal state. Our results showed that the protein levels of USP14 and SEL1L, both involved in ERAD, were significantly higher in Sh cells whereas a similar trend existed for PARN and XPOT (Fig. 1f). Thus Galectin-3, as a non-conventional RBP, modulates selectively the half-life, levels and translation of mRNAs whose encoded proteins share similar biological functions (RNA regulons).

### Localisation of Galectin-3 at the ER-mitochondria interface

**Fig. 2:**
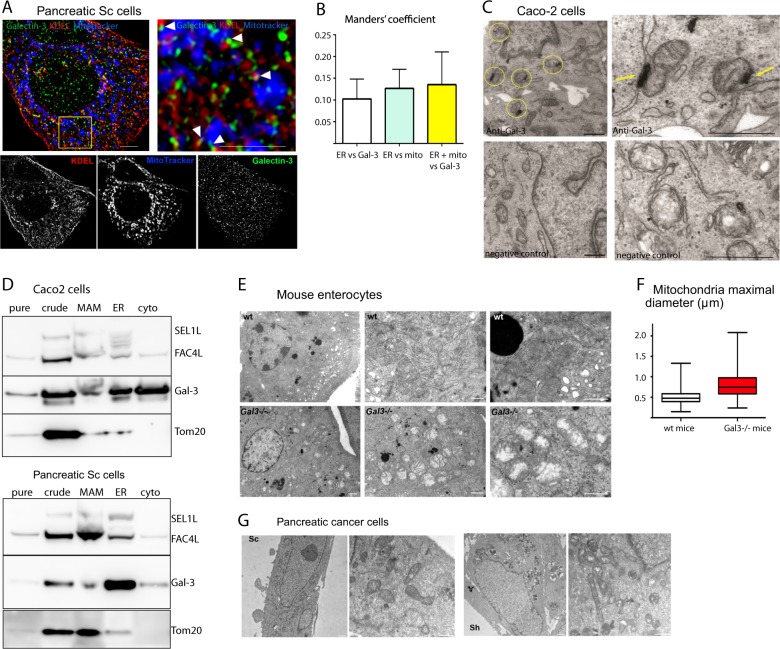
Galectin-3 localises at contact sites between the ER and mitochondria and influences mitochondria morphology. **a** 3D-Structured Illuminated Microscopy (SIM) analysis of the distribution of DsRed-KDEL (red), GreenMitoTracker (blue) and Galectin-3 (green) in control Sc cells. Scale bars, 1 μm. **b** Quantification of co-localisation was performed using the Manders overlap coefficient. Quantification of the proportion of ER overlapping with mitochondria (ER vs mitochondria): 12.64 ± 1.2%; the proportion of the ER overlapping with Galectin-3 (ER vs galectin-3): 10.25% ± 1.2% and the proportion of ER-mitochondria contacts overlapping with Galectin-3 (*ER* + *mitochondria vs Galectin-3*): 12.53 ± 2.09%. Data are mean ± SEM. **c** Transmission electron microscopy analysis of Galectin-3 distribution in epithelial Caco2 cells. Negative control was performed with immunogold labelling without the use of primary antibody. Immunogold labelling using an anti-Galectin-3 primary antibody allowed its specific detection at the contacts sites between the ER and mitochondria (yellow circles and arrows). Scale bars, 1 μm. **d** Caco2 and Sc cells were processed to obtain purified MAM fractions followed by western blot analysis of indicated proteins (*n* = 1). *Pure* pure mitochondria, *crude* crude mitochondria, *MAM* mitochondria-associated membranes, *ER* endoplasmic reticulum, *cyto* cytosol. **e** Ultrastructural analysis of the mitochondria in enterocytes of wt (upper panel), or *Lgals3*(Gal3*)*^*−/−*^ (lower panel) mouse jejunum. Scale bars, 500 nm. **f** Statistical analysis of the mean maximum diameter of mitochondria in wt (white) and *Lgals3*(Gal3*)*^*−/−*^ (red) mouse jejunum. wt: *N* = 3 mice, *n* = 632 mitochondria; *Lgals3*(Gal)3^*−/−*^:*N* = 5 mice, *n* = 663 mitochondria. (One-way ANOVA with unpaired *t*-test, *** *P*-value <0.0001). **g** Ultrastructural analysis of the mitochondria in controls Sc (left panels) or Galectin-3 silenced Sh pancreatic epithelial cells (right panels). Scale bars, 1 μm.

Since Galectin-3 post-transcriptionally regulates several genes involved in mitochondria and/or ER functioning, we next wondered whether Galectin-3 could localise at the ER-mitochondria interface. Using high-resolution fluorescence microscopy, KDEL as a proxy of the ER network and Mitotracker to stain the mitochondria (Fig. 2a), we showed that, although the ER and mitochondria display by themselves 12% of contacts, ~13% of the ER-mitochondria interfaces associate with Galectin-3 in pancreatic Sc cells (Fig. 2b). Conversely, 4% of total Galectin-3 associates with this area (as expected for a highly expressed lectin which associates with diverse subcellular compartments^13,16^). In addition, live microscopy using Mitotracker and Gal-3 dsRed construct validated the dynamic association between Galectin-3 and the mitochondria over time (Movie S1). Immunogold staining of human intestinal Caco2 cells coupled with transmission electron microscopy (TEM) revealed that Galectin-3 does associate with the ER complex. However, this association is transient, and specifically occurs at contact sites between the ER and the mitochondria (Fig. 2c, yellow circles and arrows). Finally, we performed density gradient centrifugations to collect subcellular fractions enriched in mitochondria, MAMs and ER, according to a reference method. We confirmed that a pool of intra-cellular Galectin-3 is present in MAM-enriched fraction in both Caco2 and pancreatic Sc cells (Fig. 2d). The presence of Galectin-3 in the ER fraction could be explained by its possible contamination by lysosomes. Interestingly, among the interacting partners of Galectin-3 identified in Caco-2 cells by immunoprecipitation (IP) coupled with mass spectrometry analysis, seven (≈4% of the whole) are exclusively located at the ER membrane such as TBL2, a regulator of ER stress response, and 13 in the mitochondria such ATP5J (Table S3 and Fig. S2). To conclude, by combining several approaches we show that Galectin-3 is found at MAMs thus suggesting a dedicated function of Galectin-3 at that level.

### Impact of Galectin-3 on mitochondria morphology, dynamics and bioenergetics

**Fig. 3:**
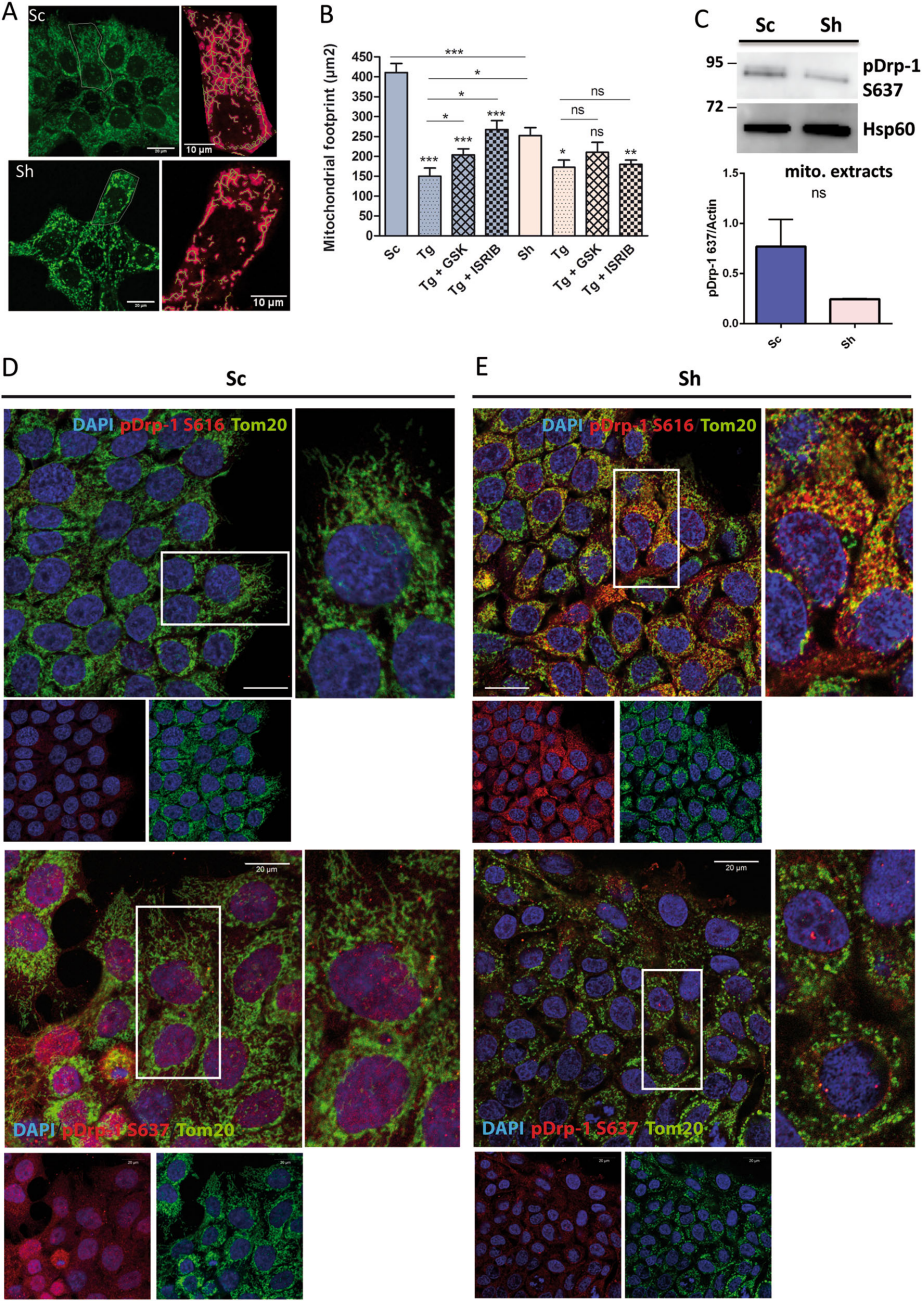
Analysis of the mitochondrial network in pancreatic Sc and Sh cells. **a** Representative images of MitoTracker staining in control Sc (top panel) and Sh (bottom panel). Cells are shown as well as the processed and skeletonised masks from which are extracted mitochondrial characterics measurement. **b** Total surface of mitochondrial network (footprint, calculated from each measured skeletonised feature in the cell) measured using the MiNA toolset from ImageJ. The macrotool was run in at least three cells isolated from acquired images from three separate experiments. ***p < 0.001 by Mann–Whitney test. *GSK* GSK2606414, *Tg* Thapsigargin. **c** Mitochondrial (mito.) extracts were analysed for the expression level of “inactive” phospho Ser637 Drp-1 by western blotting (*n* = 3). HSP60 was used as a loading marker. The fold change in phospho Drp1 levels was analysed by densitometry. **d**, **e** Representative images of control Sc (**d**) and Sh cells (**e**) immunostained for the mitochondrial marker Tom20 (green) and for “active” phospho Ser616 Drp1 (upper panels, red) or “inactive” phospho Ser637 Drp-1 (lower panels, red) and counterstained with DAPI (blue). White rectangles indicate the location of the corresponding higher magnification (right image of each panel). Scale bars correspond to 20 μm.

**Fig. 4:**
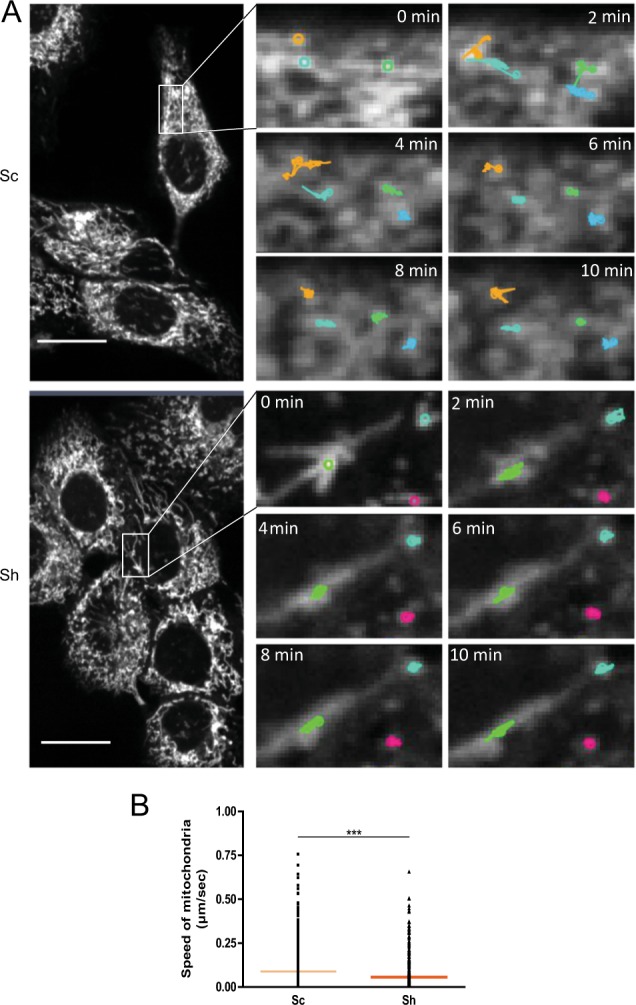
Analysis of mitochondria dynamics. **a** Time-lapse images of control Sc and Sh cells where mitochondria were labelled with Mitotracker. The open source imaging software Icy was used to detect (sensitivity: 100; 3 pixels) and track (estimated parameters) more than a hundred spots per image, each representative of isolated mitochondria. Coloured trackers are superimposed on the time-lapse images to illustrate the dynamics of each mitochondria during a 2-min time frame. **b** Speed of mitochondria was calculated for each track according to the total distance travelled by a detected spot and the duration of the frame (*n* = 3 independent experiments). Data are shown as individual values (black dots) and median (horizontal line). ***p < 0.001, Mann–Whitney test.

The ER-mitochondria interface constitutes a key hub for the ER stress response and mitochondria bioenergetics^17,18^. We then hypothesised that Galectin-3 may be instrumental there for cell adaptation to stress and/or mitochondrial homeostasis. First, we studied the impact of Galectin-3 on the morphology and behaviour of mitochondria. Ultrastructural analyses performed in WT and *Lgals3*^*−/−*^ mouse enterocytes (Fig. 2e) showed that loss of Galectin-3 provokes the formation of enlarged and swollen mitochondria whereas in wild-type cells mitochondria display the classical “stick shape”. As expected, image analysis confirmed an increased maximum diameter in Galectin-3 deficient versus control mouse enterocytes (Fig. 2f). Similarly, ultrastructural analysis of the mitochondrial network in Sh cells revealed irregular mitochondrial shape in comparison with controls Sc cells (Fig. 2g). In addition, numerous degradative compartments appear in close proximity of mitochondria in Sh cells. We concluded that Galectin-3 is required for maintenance of typical mitochondrial morphology in epithelial cells. Second, we analysed the architecture of the mitochondrial network (Fig. 3a). In dense cultures, analysis of the images with the MiNA toolset^19^ enabled to calculate the values of mitochondrial network 2D parameters. The mitochondrial footprint (Fig. 3a, b), the number of branches per network and the summed branch length (Fig S3a) were significantly decreased in Sh cells versus control Sc cells. These results show that, in the absence of Galectin-3, the mitochondrial network exhibits a less tubular shape but instead mitochondrial fragmentation (Fig. 3a, d, e). Alterations of the basal ER stress level in Sh cells could be responsible for this phenotype. Along this line, we induced acute ER stress response by treating Sc and Sh cells with 250 nM Thapsigargin alone, or in combination with either GSK2606414, a potent and selective inhibitor of PERK, or ISRIB, a PERK-independent stabiliser of eIF2α dephosphorylation. Thapsigargin in Sc cells induced a significant decrease of mitochondrial footprint (Fig. 3b), of the number of branches per network and of the summed branch length (Fig. S3), leading to mitochondrial characteristics very similar to those of Sh cells. As expected, combined treatment with GSK2606414 or ISRIB partially reversed mitochondrial morphology in Sc cells. On the contrary, in the absence of Galectin-3, treatment with Thapsigargin very negligibly altered the mitochondrial footprint and branching. Moreover, combination with GSK2606414 or ISRIB had no significant effect compared to Thapsigargin treatment alone, and did not revert mitochondrial morphology, indicating that mitochondrial fragmentation in Sh cells is independent of UPR. Hence, the loss of Galectin-3 silencing may provoke a perturbation of the mitochondrial dynamics, most probably in relation with fission/fusion events. Therefore, we analysed the behaviour of DRP-1 which is the central modulator of mitochondrial fission^20^. Phosphorylation of Drp-1 at Ser637 prevents its translocation from the cytosol to the mitochondria and its fission activity. By contrast, pSer616 Drp-1 stimulates mitochondrial fission^21^. Here we observed by immunofluorescence that pSer637 Drp-1 expression was higher in Sc than Sh cells, a trend further confirmed by western blotting analysis of mitochondrial extracts (Fig. 3d, e). By contrast, pSer616 Drp-1 was highly expressed in Sh cells and frequently co-localised with Tom20, a mitochondrial marker. Total Drp-1 level was also significantly increased in Sh cells (Fig. S3b). Altogether, these data testify a mitochondrial pro-fission phenotype in the absence of Galectin-3. Moreover, to test mitochondrial plasticity, we performed live cell imaging and mitochondrial motility was assessed by particle-tracking analysis of Mitotracker-labelled mitochondria in Sc (Movie S2) and Sh cells (Movie S3). Whatever the parameter used (i.e. speed or distance), the motility of mitochondria was significantly decreased in absence of Galectin-3 (Fig. 4a, b). Altogether, these results reveal that Galectin-3 influences the mitochondrial dynamics/homeostasis by inhibiting the fission process and promoting the motility instead.

**Fig. 5:**
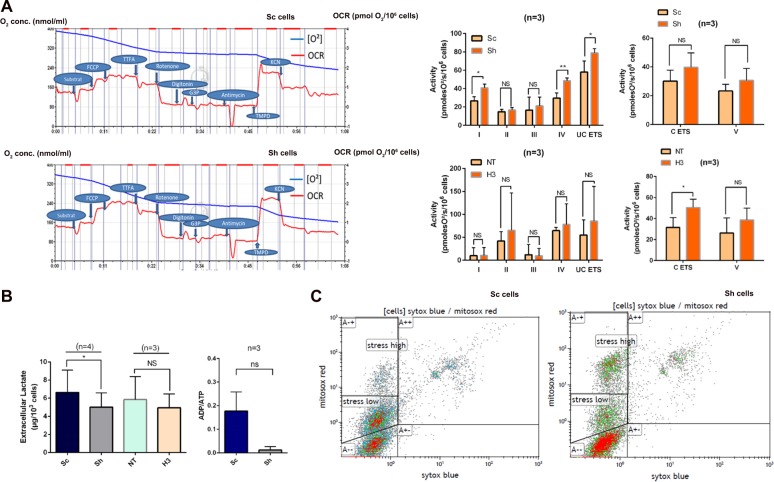
Impact of Galectin-3 expression on the OXPHOS in pancreatic and colonic control (Sc, NT) and galectin-3 deficient (Sh, H3) cancer cells. **a** The rates (red traces) of mitochondrial O_2_ consumption (blue traces) by complexes I–IV and isolated complex V were measured as described in material and methods. Left part: representative diagram of O_2_ consumption measurements from three independent experiments in Sc (upper part) and in Sh cells (lower part). Right part: Activity of the mitochondrial complexes. Upper part: pancreatic cancer cells. Lower part: colonic cancer cells. Data are means ± SEM of *n* = 3 experiments. *ETS* electron transfert system, *NS* non significant, *p < 0.05, **p < 0.01. **b** Evaluation of glycolytic activity by measurements of lactate levels in extra cellular media of pancreatic (left panel) and colonic (right panel) cancer cells, and of energetic status by measurements of intra-cellular ADP/ATP levels in pancreatic cancer cells. Data are mean ± SEM of *n* ≥ 3 experiments. **c** Dot-plots of flow cytometric analysis of the Red MitoSox™ (mitochondrial superoxide)-labelled and Blue Sytox™-labelled (dead cells) control Sc and Gal-3 deficient Sh cells. Horizontal line illustrates the intensity level used to segregate MitoSox low and high populations (representative profiles from three independent experiments).

To determine whether morphological abnormalities may impinge on mitochondrial activity, the cell respiration was studied by oxygraphy using O2k (OROBOROS instruments). Results showed that the activities of mitochondrial Complex I (NADH dehydrogenase) and Complex IV (cytochrome C oxydase) were significantly higher in Sh versus control pancreatic Sc cells (Fig. 5a). The activities of Complex II and III did not differ between Sh and control Sc cells. The electron transfer system (ETS) measured after addition of FCCP, an uncoupling agent, was significantly higher in Sh than control Sc cells (Fig. 5a). By contrast no significant difference was observed for Complex V nor ETS measured in coupled conditions (C ETS). In intestinal cells, no significant differences in the activities of Complexes I–V and ETS were observed between H3 and NT control cells, probably because of the high SD values. However, despite the versatility of Caco2 cells, Galectin-3 depleted cells presented a reproducible and significant increase in C ETS when compared to control cells (*p* = 0.0169).

Since oxidative phosphorylation (OXPHOS) is the main contributor to ATP cellular production and occurs downstream of the glycolysis, we hypothesised that this inhibitory effect of Galectin-3 may induce a cellular metabolic stress. We therefore measured extracellular lactate levels as a proxy of the glycolytic activity. Lactate levels were higher in Sc versus Sh pancreatic cells (Fig. 5b, *p* < 0.05), whereas no difference occurred between Galectin-3-silenced Caco2 cells and parental cells. However, the ADP/ATP ratio, which is inversely related to the cell energy status, was not significantly lower in Sh than control Sc cells. Thus, in the presence of Galectin-3 the mitochondrial OXPHOS is less efficient but no metabolic stress is observed. Leakage of electrons from the mitochondrial electron transport chain results in the generation of reactive oxygen species (ROS) mainly at the level of complexes I and III^22^. By flow cytometric analysis of MitoSOX® Red-stained populations, we herein showed that the number of cells with positive mitochondria was not different between Sc and Sh cells (Figs. 5c and S4). However, there was a higher percentage of high ROS cells in Sh than in control Sc cells (44% vs 16.5%, *p* < 0.0001) in agreement with a higher OXPHOS activity in the mitochondria of Galectin-3 silenced cells.

To explain these differences in mitochondria dynamics and bioenergetics, we wondered whether Galectin-3 might affect the expression of components of the respiratory chain post-transcriptionally. In this respect, we identified two species of interest among the mRNAs destabilised by Galectin-3 (Table S1). First, *PDSS2* which encodes DLP1, a protein involved in the biosynthesis of coenzyme Q which promotes electron transfer between complexes II and III^23^, and *GUF1* alias *EF4* which encodes a protein of the mitochondrial inner membrane important for complex IV organisation and activity^24^. By western blotting (Fig. S5), we showed that Galectin-3 did not influence the expression of DLP1 or GUF1. The same was observed for ISCU, a scaffold protein involved in the assembly of Fe–S cluster found in complexes I, II and III. However, among the interacting partners of Galectin-3 identified by co-immunoprecipitation we did find ICSU, and also other members of mitochondrial complexes such as UQCRC2 (Complex III) or the F0 complex of ATP Synthase (Table S3).

### Gal-3 selectively controls mRNA translation through mTORC1

**Fig. 6:**
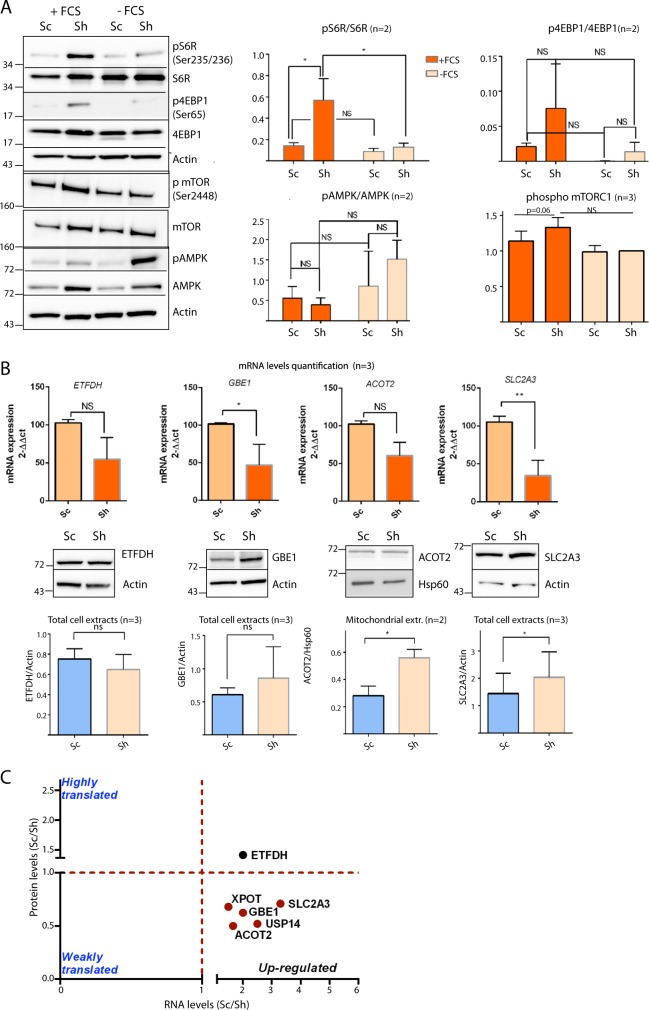
Impact of Galectin-3 on mRNA translation in pancreatic cancer cells. **a** Influence of the addition of fetal calf serum (FCS) in serum-starved control Sc cells and galectin-3 deficient Sh cells on the mTORC1 and AMPK pathways. Protein lysates were analysed for indicated proteins by western blotting. Fold change in protein levels was analysed by densitometry and Student’s *t* test. For phospho mTOR, levels are expressed relative to those in Sh cells without FCS which were set at 100%. **b** ETFDH, GBE1, ACOT2 and SLC2A3 mRNA and protein levels were evaluated simultaneously by Q-PCR (*n* = 3) or western blotting (*n* ≥ 2) in control Sc or galectin-3 deficient Sh cells. Fold changes are shown on corresponding graphs. **c** Schematics showing classification of mRNAs according to the influence of Galectin-3 on mRNA and protein expression: blocked in mRNA translation (red dots) and unaffected (black dot).

Since mTORC1 is a global sensor of the cell nutrient status we wondered whether Galectin-3 expression may influence mTORC1 activity due to its negative impact on OXPHOS. Addition of fetal calf serum (FCS), an activator of mTORC1, in serum-starved cells failed to induce S6RP and 4EBP1 phosphorylation in control cells by contrast with Sh cells (Fig. 6a). In agreement, a trend towards higher levels of phosphorylated mTOR at Ser2448 was observed in Sh cells vs control cells but the phospho-mTOR/mTOR ratio was not different between Sh and Sc, probably because a decrease of total mTOR occurred in Sc cells (Figs. 6a and S6). No difference was observed for the AMPK pathway between Sc and Sh cells, in accordance with the absence of metabolic stress reported earlier (Fig. 5b). Thus the presence of Galectin-3 decreases S6RP and 4EBP1 activity in part by reducing mTORC1 activation. Since phosphorylation of both S6RP and 4EBP favours mRNA translation initiation, Galectin-3 inhibitory effect may induce a blockage of mRNA translation in pancreatic cancer cells in usual culture conditions (with FCS). To validate this point, we prepared protein extracts and total RNA simultaneously and measured the protein levels by western blot and mRNA levels by qPCR of four genes representative of the mTORC1 and fatty acid metabolism GO categories whose mRNAs are stabilised by Galectin-3. Whereas *GBE1* and *SLC2A3* mRNA levels were higher in Sc cells than in Sh cells, the corresponding protein levels were unchanged or decreased in Sc versus Sh cells, respectively (Fig. 6b). Similarly, ACOT2 protein levels were decreased in Sc mitochondrial extracts whereas its mRNA levels were similar between Sc and Sh cells. Thus, the differential expression of transcript and protein observed for *GBE1, ACOT2* and *SLC2A3* but also for USP14 and XPOT (Fig. 1f) suggests that, in presence of Galectin-3, the translation of a fraction of these 5 mRNAs is blocked (Fig. 6c). A second category of mRNAs is represented by *ETFDH* whose mRNA and protein levels were unaffected by Galectin-3 expression (Fig. 6). In conclusion, Galectin-3 appears to reprogram selectively the translatome of pancreatic cancer cells in basal conditions through the control of S6RP and 4EBP1 activity.

### Gal-3 promotes an early transactivation of ATF6- and Xbp-1s target genes following ER stress induction

**Fig. 7:**
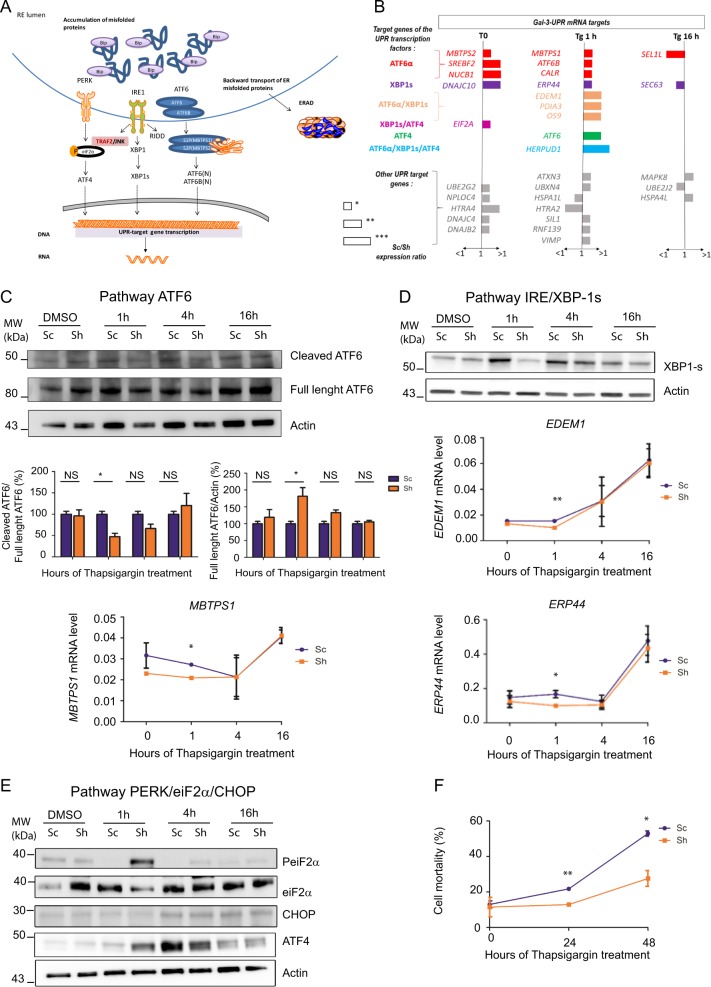
Impact of Galectin-3 on the ER stress response. **a** Schematics showing the three branches activated by the accumulation of misfolded proteins in the ER lumen. **b** Expression levels of 84 genes related to the UPR pathway were analysed by real-time qPCR in control Sc and Sh cells at baseline and 1 h, 4 h and 16 h after addition of Thapsigargin (250 nm) (*n* = 2 independent experiments). Genes whose mRNA levels were significantly different between control Sc and Sh cells are presented in the graph and classified into four subgroups according to their dependence towards ATF6α (in red), XBP-1s (in purple), ATF6α and or XBP-1s (in orange), ATF4 or XBP-1s (pink), ATF4 (green), ATF6α or ATF4 or XBP1s (light blue). This classification relies on the analysis of promoter cis element structures (ERSE and UPRE) of the UPR-related genes performed elsewhere by Montibeller et al., using JASPAR, REACTOME, and TRUSST databases. Expression level is shown as the Sc cells mRNA levels/Sh cells mRNA levels ratio. The length of the graph bar is related to the level of statistical significance of variation. **c** ATF6 pathway activation: Variation of full length and cleaved ATF6 proteins (upper panel) during Thapsigargin treatment studied by western blotting (*n* = 2 independent experiments). Fold change in protein levels was analysed by densitometry (medium panel). Expression level was arbitrarily set at 100% for Sc cells. Data are shown as mean ± SEM and were compared by Student’s *t* test. Variation of the mRNA levels of *MBTPS1*, an ATF6-N target gene, was followed by real-time qPCR both at baseline and during Tg treatment (*n* = 2, lower panel). **d**. IRE/Xbp-1s activation: variation of XBP-1s protein levels (upper part) by western blotting (*n* = 3) and variation of the mRNA levels of *EDEM1* and *ERP44* (two XBP1s targets, lower part) was followed by real-time qPCR both at baseline and during Tg treatment (*n* = 2). **e** PERK activation: variation of phospho-eIF2α, ATF4 and CHOP proteins levels (upper part) by western blotting (*n* = 2). **f** Cell mortality during a long term Thapsigargin treatment was assessed by cell cytometry with the Live and Dead Assay. Data are shown as mean ± SEM of two independent experiments and were compared by Student’s *t* test (*p < 0.05; **p < 0.01; ***p < 0.001; ns non significant).

In mammalian cells, three canonical ER resident transmembrane proteins (IRE1, PERK and ATF6) act as ER stress sensors and activate three pathways (Fig. 7a). mRNA expression levels of 84 key UPR genes were then monitored by PCR arrays, at baseline (T0) and 1, 4 and 16 h following ER stress induction by treatment with 250 nM Thapsigargin. Levels of 31 mRNAs showed significant variations, among which 26 were significantly increased at early phases of cell response to Thapsigargin treatment whereas 5 were diminished in control Sc cells versus Sh cells respectively (Fig. 7b). Most of these mRNA inductions in Sc cells occurred at steady state (38.5%) or after 1 h of Thapsigargin treatment (61.5%). Based on promoter structure analysis^25^, the 31 mRNAs which showed differential expression between Sc and Sh cells were classified according to the specific contribution of ATF4, ATF6(N) and Xbp-1s transcription factors for their expression (Fig. 7b). Interestingly, seven mRNAs correspond to ATF6(N)-target genes, three mRNAs to Xbp-1s-target genes, three mRNAs to ATF6(N) and Xbp1-s target genes, whereas only one mRNA corresponds to ATF4-target genes. Next we decided to study the impact of Galectin-3 on the three UPR branches at the protein level. As soon as 1 h of Thapsigargin treatment, the ratio cleaved ATF6/full length AT6 protein was significantly increased in control Sc cells, demonstrating an engagement of the ATF6 arm (Fig. 7c). Hence, we asked whether this could translate into early transactivation of specific ATF6 targets. Indeed, we observed that 1 h after Thapsigargin treatment *ATF6B and MBTPS1* mRNA levels (Figs. 7c and S7) were significantly increased in Sc versus Sh cells. Regarding the IRE1 branch (Fig. 7d), Xbp-1s protein expression was increased in control Sc cells as early as 1 h after Thapsigargin addition (*p* < 0.05 versus baseline) whereas in Sh cells activation was delayed. Maximal induction of Xbp-1s occurred at 4 h time course in both cell lines (Figs. 7d and S7). Accordingly, some XBP-1s-dependent targets such as *ERP44, EDEM1 (*Fig. 7d*)* or *HERPUD1* (Fig. S7) were also significantly up-regulated in control Sc cells 1 h after Thapsigargin treatment. Finally, concerning the PERK arm (Fig. 7e), phosphorylation of eif2α was delayed in control Sc cells as was ATF4 protein expression. In fact, ATF4 protein levels were significantly higher as early as 1 h after Thapsigargin addition only in Sh cells (*p* < 0.05 versus baseline, Fig. S5). Since *ATF4* mRNA levels did not vary significantly during Thapsigargin treatment, ATF4 protein expression results from a phospho-eif2α-dependent translation reprogramming. Taken together, these data show that Galectin-3 favours an early activation of the ATF6 and IRE/Xbp1s arms of the UPR resulting in an increase of the ER protein folding (*ERP44, CALR)* as well as ERAD pathway (*HERPUD1*) capacities. Moreover, Galectin-3 also delays ATF4 expression during an experimental ER stress. Finally, we studied the influence of Galectin-3 on cell viability during a prolonged Thapsigargin treatment (>16 h). By live and dead assay, we demonstrated that the mortality of control Sc cells was significantly higher than those of Sh cells at 24 and 48 h of the treatment (Fig. 7f).

## Discussion

Our data showed that Galectin-3 behaves as a selective post-transcriptional regulator which coordinates the expression of 13 RNA regulons which merge subsets of functionally related mRNAs^26^. Some RNA regulons control a biological function previously linked to Galectin-3, such as cell death/apoptosis^27^ or protein secretion^28^, which strengthens the validity of our experimental approach. Interestingly, other RNA regulons such as fatty acid metabolism, cholesterol homeostasis or UPR were previously rarely or never related to Galectin-3. We thus focused our work on Galectin-3’s control of the mitochondrial bioenergetics and the ER stress response in epithelial cancer cells.

Regarding the mitochondrial function, our data demonstrated a more efficient OXPHOS in the absence of Galectin-3, and especially a higher activity of complex I (NADH oxidase) and complex IV (cytochrome C oxidase) in pancreatic cancer cells. The activity of complex V (ATP synthase) was increased in the absence of Galectin-3 but not significantly. How does Galectin-3 affect the activity of the mitochondrial OXPHOS complexes? Despite its ability to inhibit the mTORC1/S6RP-4EBP1-dependent translation, we did not highlight any impact of Galectin-3 on the protein levels of key regulator of OXPHOS such as DLP1^23^, GUF1/EF-4^24^ or ISCU, a scaffold protein important for the assembly of Fe–S clusters found in Complexes I, II and III^29^. Previous^15^ and our immunoprecipitation data demonstrated however that Galectin-3 interacts with the F1^15^ and F0 subunits of ATP synthase located in the mitochondria inner membrane and also ICSU, thus suggesting that Galectin-3 may prevent the assembly of mitochondrial complexes and finally decreases OXPHOS. An alternative hypothesis consists in considering that Galectin-3’s impact on OXPHOS is linked to its regulator role on the ER-mitochondria communication. Indeed, in normal and cancer epithelial cells deriving from the digestive tract, Galectin-3 is localised at the interface between ER and outer mitochondrial membrane. Moreover we confirmed by subcellular fractionation experiment the presence of Galectin-3 within MAMs, in agreement with previous proteomic studies conducted on human fibroblasts^30^ or on mouse brain^31^. This is probably not random since MAMs are enriched in proteins involved in mitochondrial function or Ca^2+^ trafficking such as the calcium channels InsP3Rs at the ER membrane or VDAC1 on the mitochondria outer membrane^31,32^. Therefore, the inhibitory effect of Galectin-3 on OXPHOS may result from its ability to perturb calcium exchanges at MAMs which are crucial for mitochondrial bioenergenetics^32,33^.

Regarding mitochondrial morphology, we observed profound alterations of the mitchondrial network and dynamics in absence of Galectin-3 with more fragmented and round mitochondria. Different mechanisms may participate in these changes. First, Galectin-3 localised at MAM, may prevent the wrapping of the ER around mitochondria which occurs during the first step of mitochondrial fission^2,34^. Accordingly, the “pro-fission” phospho-Ser616 Drp1 was increased and recruited to the mitochondria in absence of Galectin-3. Second, the alterations of the mitochondrial network observed in Sh cells are reproduced by Thapsigargin treatment of Sc cells. Thapsigargin, a SERCA inhibitor, is known to induce mitochondrial fragmentation by increasing mitochondrial Ca^2+^^ 35^. By constrat, PERK silencing normalises mitochondrial Ca^2+^ level and rescues mitochondrial morphology^36^. Thus, we proposed that Galectin-3 controls the Ca^2+^ exchanges at MAMs between the ER and mitochondria^37^ and prevents mitochondrial Ca^2+^ overload in cancer cells. This point will require additional investigations.

Considering the ER per se, no significant differences in the activity of the three arms of the UPR, notably the PERK /ATF4 branch involved in the control of mitochondrial respiration^38^ occurred between Sc and Sh cells in basal conditions. Therefore, an involvement of the ER in the effects of Galectin-3 on mTORC1 or OXPHOS can be excluded. However, after induction of an acute ER stress, Galectin-3 favours an early activation of the IRE/Xbp-1s and ATF-6 branches of the UPR and delays those of the PERK/ATF4 pathway resulting in an adaptative response. However, when the stress became chronic (>16 h), Galectin-3 triggers cell death, which corresponds to the proper UPR response occurring when the adaptative phase fails.

**Fig. 8:**
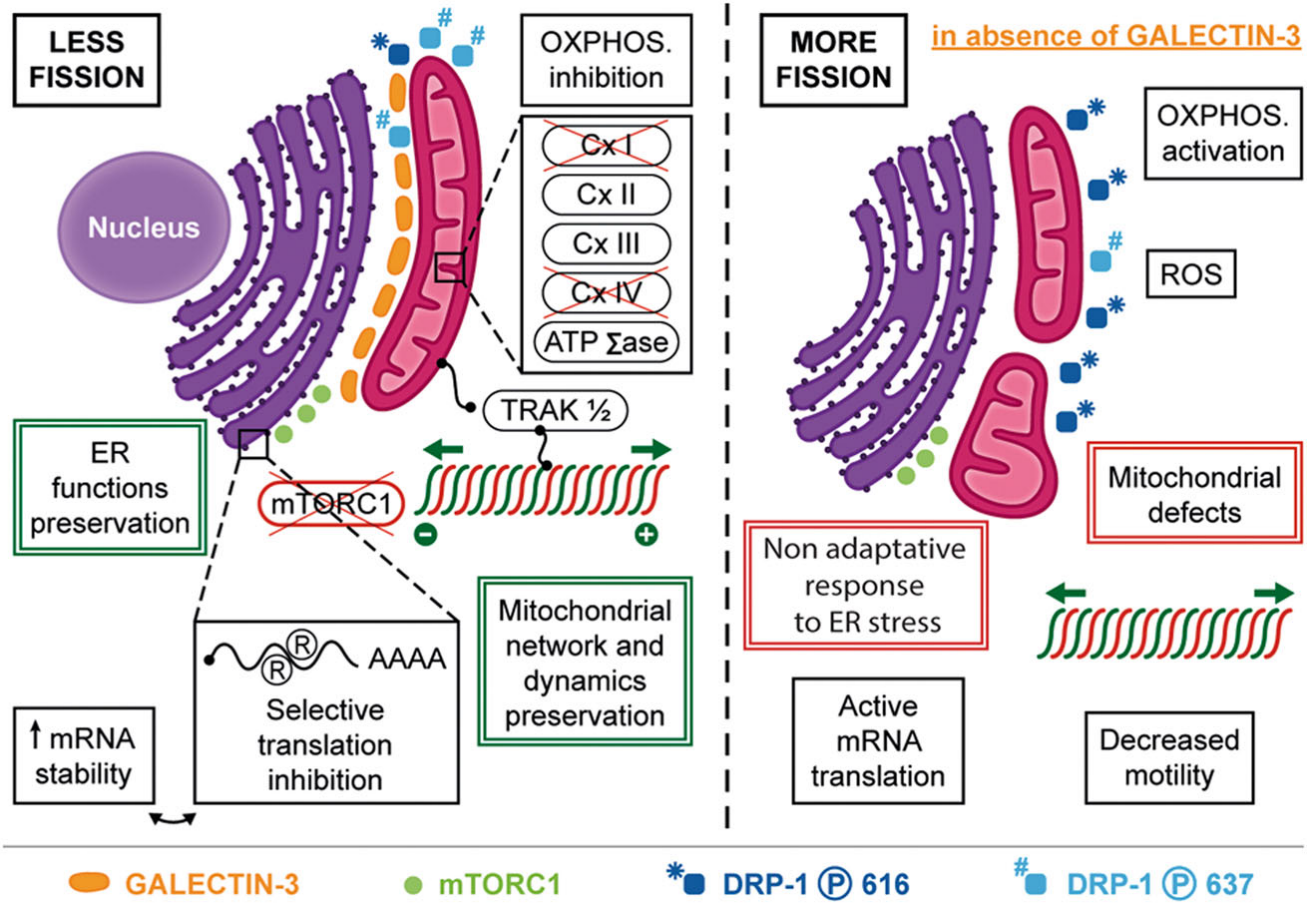
Schematics recapitulating the different impacts of Galectin 3 expression on mitochondria and ER functioning. At the ER-mitochondria interface, Galectin-3 (i) prevents excessive mitochondrial fission and preserves the shape and dynamics of the mitochondrial network; (ii) modulates OXPHOS activity; (iii) reprograms selectively the mRNA translation via a partial inhibition of the mTORC1 pathway; (iv) favours an adaptative UPR following ER stress induction.

To summarise, we show that the localisation of Galectin-3 at MAM is accompanied by novel functions for this lectin. At the ER, Galectin-3 promotes an earlier activation of two branches of the UPR which result in an adaptative response to ER stress. Simultaneously, Galectin-3 preserves the mitochondrial network and dynamics independently of the UPR and regulates cell bioenergetics (Fig. 8). Therefore, Galectin-3 appears to act as a coordinator of the ER and mitochondria functioning.

## Materials and methods

### Cell culture

Caco2_BBE_ cells were routinely grown in DMEM 4.5 g/l glucose, 20% foetal bovine serum, 10% penicillin-streptomycin (Gibco, Thermo Fischer Scientific, Waltham, MA). The culture medium was renewed every day. Galectin-3 reduction was carried out by lentiviral delivery of shRNA constructs directed against human *LGALS3* (*shGalectin-3*: TRCN0000029305 5′-CCGGCCCACGCTTCAATGAGAACAACTCG-AGTTCTCATTGAAGCGTGGGTTTTT_3′) designed and cloned into the lentiviral pLKO.1 puromycin resistant vector MISSION shRNA lentiviral Transduction particle (Sigma Aldrich). Clone generation was performed in DMEM supplemented with 20% FBS, 10% penicillin/streptomycin and 2 μg/ml puromycin (Invivogen, San Diego, CA) and selected clones were routinely maintained in 1 μg/ml puromycin. Control Caco2 clones (*shNT*) were generated using pLKO.1-puro non-target shRNA control transduction particles SHC016V (5′-CCGGGCGCGATAGCGCTAATAATTTCTCGAGAAATTAT-TAGCGCTATCGCGCTTTTT-3′). Efficiency and specificity of Galectin-3 reduction was assessed by Western blot (Fig. S1). Control Sc cells and Galectin-3 knock-down cells (Sh1, Sh2 and Sh3) were obtained by stable transfection of human pancreatic cancer cells T3M-4 with pSuper.Retro.puro vectors, as previously described^39^. The Sh1 cells were used throughout the study (and called Sh thereafter) since they exhibited a high silencing of *LGALS3* (Fig. S1). Sc and Sh cells were routinely grown in RPMI 1640 medium supplemented with 15% of FCS, 2 mM L Glutamine, 1% penicillin-streptomycin and 200 ng/ml puromycin (Gibco). The cells were authenticated by ATCC using STR profiling. For ER stress experiments, cells were treated with 250 nM of Thapsigargin alone, or combined with 250 nM or IRSIB or 250 bM of GSK2606414 for 4 h. concentrations of inhibitors were determined by evaluation their impact of Thapsigargin-induced phophorylation of eIF2α (not shown). DMSO was used as a control.

### Mice

Wt and Gal-3 null mutant (*Lgals3*^*−*/*−*^) mice of the 129/Sv background^40^ were housed in EOPS (environment without specific pathogenic organisms) environment and handled in accordance with the French regulation for animal care. Experimental protocol was approved by the ethical committee of the Paris Diderot University (approval #A75-13-17).

### qPCR

Total RNA was extracted using “Nucleospin RNA II” from Macherey Nagel. RNA concentration and purity were determined with Nanodrop. Quantification of Gal-3 targeted mRNAs was carried out by qRT-PCR (relative quantification, Taqman technology) using the 2^*−*ΔΔCt^ method and *GAPDH* as an internal standard. Each sample was run in triplicate. Primers are available in Table S4. UPR gene analysis was performed with RT2 Profiler PCR Array System (Qiagen, Courtaboeuf, France) which contained 84 genes for the UPR pathway (Cat.# PAHS-089Z). To this purpose cells were treated with thapsigargin (250 nM, Sigma Aldrich, Lyon, France) for 1 h, 4 h and 16 h or with DMSO as control. RNAs were collected and extracted as previously described^7^. Reverse Transcription was carried out with the RT2 First Strand Kit (Qiagen) following the manufacturer’s instructions. Quantitative Real-Time PCR was performed on QuantStudio 7 Flex Real-Time PCR System (Life technologies). Standard 2 ^*−*ΔΔCt^ method was used for determining changes in gene expression according to genes used as references in the kit. Jaspar reactome is an open-access database for eukaryotic transcription factor binding profiles experimentally defined^41^.

### Western blot

Western blot were performed as previously described^7,39^. Membranes were incubated overnight at 4 °C with specific antibodies as detailed in (Table S4). The membranes were then incubated with peroxydase-conjugated secondary antibodies (Sigma-Aldrich) and revelation was performed with LAS 4000 (Fujifilm) using the West Pico chemoluminescent substrate (Perbio, Villebon/Yvette, France). Mitochondria extracts were prepared using the Mitochondria Isolation Kit (from Pierce, ThermoFisher, France) according to the manufacturer’s instructions.

### OXPHOS

Respirometry experiments were performed at 37 °C in the two glass chambers of an OROBOROS Oxygraph under normoxic conditions. DatLab software (OROBOROS INSTRUMENTS, Innsbruck, Austria) was used for real-time data acquisition and analysis. Experiments were carried out according to a previously published protocol^42^ with some modifications. In brief, cells were seeded onto T75 culture flasks and culture for 7 days to reach ~80% confluence. Cells were trypsinized, washed with PBS and resuspended in mitochondrial respiration medium (MiR05: 110 mM sucrose, 60 mM K ^+^-lactobionate, 0.5 mM EGTA, 3 mM MgCl_2_, 20 mM taurine, 10 mM KH_2_PO_4_, 20 mM HEPES, 1 g/L BSA fatty acid free and adjusted to pH 7.1 with KOH at 37 °C) to obtain a concentration of 3.0 × 10^6^ cells per mL. Subsequently, basal OXPHOS was measured after adding glutamate (10 mM), pyruvate (10 mM), malate (10 mM) and succinate (10 mM). Complexes I to IV were studied together in three different series whereas complex V was studied isolated in three different series. Uncoupling was performed by stepwise titration of carbonyl cyanide p trifluoromethoxy phenyl hydrazone (FCCP 1 μM 0.5 µl/0.5 µl steps), followed by CII inhibition (thenoyltrifluoroacetone (TTFA, 50 µM) and then CI inhibition (rotenone, 2.5 µM). CIII was inhibited after addition of sn-glycerol-3-phosphate (10 mM) by antimycin A (2.5 μM) to correct for residual oxygen consumption, followed by N′-N′-tetramethyl-p-phenylenediamine (TMPD, 300 µM) adding and cyanide potassium (KCN, 2 mM) to inhibit CIV.

For the study of Complex V, the substrates were introduced at the same concentration as previously. Cell permeabilization was then carried out directly in the chamber by digitonin (8.1 mM, 1 µl/million of cells). Then ADP (2.5 mM) was added to exhibit phosphorylation, followed by CV inhibition (oligomycin, 2.5 μM), uncoupling by FCCP (1 μM 0.5 µl/0.5 µl steps), inhibition by KCN (2 mM) and antimycin A (2,5 µM) to correct for residual consumption. Respiratory flow, Oxygen Consumption Rate (OCR), is expressed as pmol O_2_·s^*−*1^·10^−6^ cells.

### ATP and lactate assays

ATP levels in cells were determined by using the ADP/ATP ratio assay kit (Sigma-Aldrich, MAK135) according to the manufacturer’s protocol. Briefly, Sc and Sh cells were seeded (5000/well) in a 96-well, flat-bottom, white plate with clear bottoms (Greiner Cellstar®) and grown in medium described above for 48 h. Luminescence was read in a Mithras LB 940 luminometer. All analyses were performed in triplicate. Cells were seeded onto T25 culture flasks and culture for 5 days to reach ~80% confluence. Then 0.5 ml of medium was removed from each culture and deproteinated by adding 0.5 ml of perchloric acid at 8% v/v, vortexing for 30 s, then placing the mixture in 4 °C for 5 min, and centrifugating at 4500×*g* for 10 min. Accumulation of lactate in the culture medium was determined using lactate assay kit (Lactate Gen.2 Roche). To determine lactic acid, 500 µl of the deproteinated supernate was added to a reaction mixture containing B reagent (Hydrogen donor: 1,75 mmol/L, ascorbate oxidase: 501 µkat/L) and C reagent (4-Aminoantipyrine: 5 mmol/L, lactate oxidase: 251µkat/L, peroxidase: 401 µkat/L). Formation of lactate was measured by a Konelab 60 analyzer. Samples were analysed in quadruplicate (Sc/Sh) and triplicate (NT/H3).

### Reactive oxygen species

To assess Reactive Oxygen Species (ROS) levels, the MitoSox Red reagent was used ((Molecular Probes, Thermo, Illkirch, France). It is selectively captured by mitochondria, and oxidised by superoxyde anion before exhibiting a red fluorescence. Cell viability was assessed using Sytox Blue reageant (Molecular Probes) which labels cells exhibiting a loss of membrane integrity. Three independent experiments run in triplicate were performed. Each experiment includes a negative control (unlabelled cells) and a positive control where cells were cultured in presence of 100 microM menadione, an inducer of oxidative stress, during 90 min before sample preparation. Sample Preparation: After trypsinization, cells (300,000) were incubated with MitoSox Red reagent (2.5 microM, 30 min, 37 °C) in cytometry-specific tubes. Cells were then pelleted by centrifugation (1500 rpm, 5 min), the supernatant was discarded and the cells washed once in PBS buffer. Then, cells were labelled by Sytox blue(5 microg/ml) during 10 min at RT in a dark room. Reaction was stopped by placing the tubes in ice before being analysed by Cell cytometer on a Becton Dickinson LSRFortessa X-20 using Kaluza software (Beckman coulter, Villepinte, France).

### Live cell imaging

Sc and Sh cells were grown in µ-slide 8-well dishes (Ibidi) for 24 h before transfection with Gal-3 dsRed construct (king gift of Pr R. Jacob, Marburg^43^) as previously described^44^ and adapted to 8-well µ-slides. After 48 h, cells were rinsed with 1X HBSS buffer and then incubated for 30 min at 37 °C in 1X HBSS buffer containing 250 nM MitoTracker™ Green FM (Invitrogen™, Thermo, Illkirch, France). Cells were maintained at 37 °C and 5% CO2 during real-time acquisitions. Ten-minute time-lapse acquisitions were performed using an inverted Yokogawa Spinning Disk confocal microscope with a 100× oil-immersion lens (NA 1.4 with an optical resolution of 164 nm, Zeiss). Images were acquired and processed with ZEN software for further analyses with the open source image processing software Icy. All setups, using similar illumination and recording conditions (detector frequency, gain and laser intensity), were applied to Sc and Sh cell lines.

### Immunofluorescence and SIM microscopy

Cells were seeded at 20,000 cells per 8-well Lab-Tek chamber slides (Nunc) and culture for 72 h. Cells were then stained using 250 nM Mitotracker Green FM for 30 min at 37 °C. Cells were rinced in 1X warm PBS with MgCl_2_ and CaCl_2_ and were fixed in 2% paraformaldehyde in 1X PBS pH 7.4 for 10 min. After permeabilization and saturation for 20 min respectively with 0.025% saponin and 0.025% saponin and 1% BSA in 1X PBS, anti-Galectin-3, anti-pSer637-Drp-1 or anti-pSer616Drp-1 antibodies was added at a 1:50 (Galectin-3) or 1:100 final dilution in 0.025% saponin, 1%BSA, 1X PBS overnight at 4 °C. Alexa488-anti-rabbit antibodies (Invitrogen) were then used as a secondary antibody.Imaging was performed on a Zeiss LSM 710 confocal microscope (Jena, Germany) with a ×63 oil-immersion lens (NA 1.4 with with an optical resolution of 164 nm).

3D-Structured Illumination Microscopy (SIM) was performed on a Zeiss Elyra Microscope coupled to an optovar 1.6, ×63 objective and a camera EM CCD Andor SIM. During z-astack acquisitions, 5 rotations were applied. Deconvoluted structured illumination images were generated by Zen software, and images were merged in ImageJ. Triple colocalization was analysed using a Matlab-based custom program. Briefly, fluorescence was first segmented in each channel using local thresholding (Phansalkar method^45^), and a local 2-D median filter with user-defined neighbourhood size was applied to remove noise. Colocalization was then measured using the Manders split coefficients M1 and M2^46^ as such:

$M1 = \frac{{\mathop {\sum }\nolimits_i S1_{i,coloc}}}{{\mathop {\sum }\nolimits_i S1_i}}$ and $2 = \frac{{\mathop {\sum }\nolimits_j S2_{j,coloc}}}{{\mathop {\sum }\nolimits_j S2_j}}$, where S1_i,coloc_ = S1_i_ if S1_i_ > 0 and S2_i_ > 0, and S2_j,coloc_ = S2_j_ if S2_j_ > 0 and S1_j_ > 0.

The Matlab Code or the standalone user interface can be shared upon request.

### Electronic microscopy

1 mm sections of wt or *Lgals3*^*−*/*−*^ mouse jejunum were fixed in 2.5% glutaraldehyde (Electron Microscopy Sciences (EMS), Hatfield, PA, USA) in 0.1 M cacodylate buffer (pH 7.4) (Sigma-Aldrich) for 2 h at 4 °C and then in 1% osmic acid (EMS) in 0.1 M cacodylate buffer for 1 h. Standard procedures for dehydration and embedding in Epon-Araldite (EMS) were used. Ultrathin sections were stained with uranyl acetate and lead citrate (Sigma-Aldrich) solutions before examination using a Tecnai T12 microscope (FEI, Eindhoven, Netherlands). Cells were fixed with 1 % glutaraldehyde in 0.1 M sodium cacodylate pH 7.2 overnight at 4 °C. They were post-fixed with 1% osmium tetroxide reduced with 1.5% potassium hexacyanoferrate (III) for 1.5 hours then with 1 % uranyl acetate for 45 min, both in distilled water at room temperature in the dark. After washing, tissues were dehydrated in graded ethanol solutions, infiltrated with epoxy resin and cured at 60 °C for 24 h. Sections of 70–80 nm thickness on formvar-coated grids were observed with a Tecnai T12 microscope (FEI, Eindhoven, Netherlands).

For the analysis of Galectin-3 distribution at the ultrastructural level, immunogold pre-embedding was performed. Human enterocytes Caco2 cells were fixed 3 h in 3% PFA, 0.04% glutaraldehyde, 0.04 M cacodylate buffer, pH7.4 and then permeabilized in 0.025% saponin solution. After 30 min incubation in solution A (i.e. 0.1% Tween20, 1% serum albumin, 0.45% gelatin and 0.4% glycin), the primary antibody rat monoclonal anti-Galectin-3 was incubated overnight at 4 °C at dilution 1/300 in solution A. Rat monoclonal antibody directed Galectin-3 was a gift of Hakon Leffler (Lund University, Sweden). After washes in 0.1% Tween20, PBS, the secondary antibody, 10 nm immunogold-conjugated goat anti-rat (British Biocell International) diluted 1/300 in solution A, was incubated 1 h. After washes in 0.1% Tween20, PBS, post-fixation was performed with 1 h incubation in 1.2% glutaraldhehyde, 0.1 M cacodylate pH7.4 and 5% sucrose. After washes in 50 mM glycin, PBS, then in 1% serum albumin, PBS, and then filtered H2O, signal enhancement was performed with the Gold Enhance EM Plus kit (Nanoprobes, NY). Cells were then post-fixed with 1 h incubation in 2% osmium tetroxide, 2% potassium ferrocyanide, 0.1 M cacodylate pH7.4. Afterwards, standard procedures for dehydration and embedding in Epon-Araldite (EMS) were used. Ultrathin sections were stained with uranyl acetate and lead citrate (Sigma-Aldrich) solutions before examination using a Tecnai T12 microscope (FEI, Eindhoven, Netherlands).

### Whole transcriptome stability

Total RNA was extracted from Sh1 and Sc cells cultured in the presence of 8 μg/ml actinomycin D for 0, 3, 6, 9, 12, 24 and 30 h. A TruSeq Stranded mRNA Sample Preparation Kit (Illumina Inc., SanDiego, California) was used to construct cDNA libraries according to the manufacturer’s instructions with a fragmentation time of 2 min at 94 °C. The individual libraries were multiplexed (2pools, 7 libraries/pool) and sequenced on HiSeq2500 Illumina sequencer using a High Output paired end 2*75 bp run (UMR CNRS 8199 platform, Lille). qPCR quantification and QuBit control of the pool was done before cBot clusterization. Minimal number of reads per sample was 114 × 10^6^. RNA-Seq reads were aligned to hg19 reference genome using Tophat v2.0.12^47^. Cufflinks v2.2.1 was used to assemble transcripts and derive as Fragments Per Kilobase of exon model per Million mapped reads (FPKM) values with the ucsc hg19 transcriptome as a reference^48,49^. Expression data among time points were normalised by the geometric mean of several stable transcripts: *RPLP0* (NM_001002), *PGK1* (NM_000291) and *TRAP1* (NM_016292) (Fig. S8). FPKM normalisation, decay model assessment and calculation of mRNA half-lives were conducted according to the protocol described in Imamachi et al.^50^. Statistical analyses throughout this study were performed with statistical program R^51^. To identify the Gene Ontology (GO) terms enriched in our mRNA data set we used the GSEA computational method^50^ and two reference gene sets (Hallmark and GO cellular component) from the Molecular Signatures Data base (MSigDB)^52^. For the UPR category, GSEA analysis was also performed using AmiGO, an official web-based open-source tool for querying, browsing, and visualising the Gene Ontology and annotations as described by Monoz-Torres^53,54^. RNA Seq data have been deposited in the Sequence Read Archive of the NCBI (accession number: PRJNA552473).

### Immunoprecipitation and mass spectrometry analysis

The identification of the Galectin-3 binding proteins was performed as previously described^10^. Briefly, cell extracts were precleared by incubation with Protein-A Sepharose beads (Sigma-Aldrich), and then rat monoclonal anti-Galectin-3 antibody was applied. Immunoprecipitation of EpCAM and incubation of Protein-A coated beads alone served as controls. Immunocomplexes were recovered by addition of Protein-A/Sepharose beads overnight at 4 °C, followed by three washes in PBS. To identify the co-immunoprecipitated proteins, beads were submitted to proteolytic digestion and the peptides were analysed by mass spectrometry (ESI-LTQ-Orbitrap spectrometer) coupled with nanoLC chromatographic separation system (Proxeon) in the IJM Proteomic facility (Institut Jacques Monod, Paris, Fr).

### MAM isolation

MAM were isolated from pancreatic Sc and Caco2 cells as previously described^55^. 30 μg of proteins of each fraction were analysed by western blotting. SEL1L was used as a marker of the ER, FAC4L as a marker of MAM and Tom20 as a mitochondrial protein.

### Cell viability

Cells were treated with 250 nM of thapsigargin or DMSO as a control for 24 or 48 h. Mortality and viability was assessed by flow cytometry using the Live and Dead assay (Thermofisher, USA) according to the manufacturer instructions.

### Statistical analyses

For each kind of experiment (WB, qPCR and enzymatic complex activities) *n* = 3 biological replicates were carried unless otherwise mentionned in the text. All statistical analyses were performed with the GraphPad Prism software version 6.05. Data are expressed as mean ± SEM unless otherwise mentionned. Data between 2 groups were compared using Student’s t-test with Welch correction. Multiple data sets were compared by one-way ANOVA. Frequencies were compared using the Khi2 test. Differences were considered significant if *p* values ≤0.05. *p < 0.05, **p < 0.01, ***p < 0.001 and NS is non significant.

## Supplementary information

Supplementary Figure Legends

s1

S2

S3

S4

Figure S1

Figure S2

Figure S3

Figure S4

Figure S5

Figure S6

Figure S7

Figure S8

Supplemental Movie S1

Supplemental Movie S2

Supplemental Movie S3
