# Supplementary material for: Galectin-3 modulates epithelial cell adaptation to stress at the ER-mitochondria interface
Source: Cell Death Dis. 2020 May 12;11(5):360. doi: 10.1038/s41419-020-2556-3 (PMC7217954; doi:10.1038/s41419-020-2556-3)
Supplement: Supplementary file 1 — Supplementary Figure Legends [file 41419_2020_2556_MOESM1_ESM.docx]

**Supplementary Figure Legends**

**Table S1.** List of the mRNAs whose half-life is influenced by Galectin-3 expression in pancreatic cancer cells.

**Table S2.** GSEA Analysis performed on the experimental set of mRNA stabilized/destabilized by Galectin-3 (n=440)

**Table S3.** Galectin-3 interacting proteins identified by co-IP and proteomic analysis

**Table S4.** Supplemental Materials and methods.

**Fig S1.** Analysis of the efficiency of Gal-3 silencing in pancreatic and colon cancer cells by Western Blotting

**Fig S2.** Galectin-3 interacts with TBL2. Cells lysates (250 μg) from Sc and Sh cells were immunoprecipitated with an anti-Galectin-3 rabbit polyclonal antibody (Sc-20157) or a normal rabbit IgG as previously described (ref 37). The co-IP complexes were separated by PAGE-SDS before being immunoblotted with a rabbit anti TBL 2 antibody (Proteintech).

**Fig S3.** **A.** Analysis of the mitochondrial network in pancreatic Sc and Sh cells. Mitochondrial network morphology measurement using the MiNA toolset from Image J. The macro tool was run in at least three cells each isolated from acquired images from three separated experiments. The mean number of branches and the mean length of summed branches of the mitochondrial network (calculated from each measured skeletonized feature in the cell) are represented as bar graphs. ***, p<0.001 by Mann-Whitney test. GSK: GSK2606414; Tg: Thapsigargin; Sc: Sc control cells; Sh: Sh control cells**. B.** Analysis of total DRP1 expression by western blotting in protein lysates from control Sc and galectin-3 deficient Sh cells. Fold change was determines by densitometry. Representative image of 3 independent experiments. ns, non significant , Student’s t test.

**Fig S4.** Total number of mitosox-labelled mitochondria (left panel) and repartition of mitosox-labelled mitochondria according to fluorescence intensity : high ROS for high intensity and low ROS for low intensity of the labelling (right panel). ns, non significant (paired Student’s t test, ***, p<0.0001, Khi^2^ test).

**Fig S5.** Analysis of DLP1 (*PDSS2*), GUF1 (alias EF-4) and ICSU expression by western blotting in protein lysates from control Sc and galectin-3 deficient Sh cells. Fold change was determines by densitometry. Representative image of 3 independent experiments. ns, non significant , Student’s t test.

**Fig S6.** Phospho mTOR and mTOR levels were determined after densitometric analysis of the bands separated by western blotting analysis. For each condition a phospho mTOR/ m TOR ratio was calculted. Results are shown as mean ± SEM of 3 independent experiments. Comparison were carried out by paired Student’s t test.

**Fig S7.** **ER stress response in Sc and Sh cells**

Evolution of *ATF6B, MBTPS1, HERPUD1* mRNA levels in control Sc and Sh cells during cellular stress response induced by thapsigargin treatment. mRNA levels were determined with the UPR RT² profiler PCR array after collection and extraction of total mRNAs. N=2 independent experiments; *, p<0.05, ***, p<0.001 by Student t test. Xbp-1s and ATF4 protein levels were determined by western blotting as shown in figure 8d and 8e. Fold change was determined by densitometry and Student t tests. N=3 (Xbp1) or N=2 (ATF4) independent experiments.

**Fig S8.** Decay curves of the reference transcripts chosen for the normalization of mRNA levels in control Sc and Sh cells after Actinomycin D treatment in the whole stability experiment**.** Decay curves were built using FPKM at different time of treatment in each cell line. These reference transcripts are stable in the two cell lines since their half-life is >> 24 hours.
