# Supplementary material for: Galectin-3 modulates epithelial cell adaptation to stress at the ER-mitochondria interface
Source: Cell Death Dis. 2020 May 12;11(5):360. doi: 10.1038/s41419-020-2556-3 (PMC7217954; doi:10.1038/s41419-020-2556-3)
Supplement: Supplementary file 5 — S4 [file 41419_2020_2556_MOESM5_ESM.docx]

**Table S4– Materials and methods**

**Antibodies and dyes**

| **Antibody anti** | **Provider** | **Clone/source** | **Application** |  |
| --- | --- | --- | --- | --- |
| S6RP | Cell Signaling Tech-2217 | 5G10 | WB |  |
| phosphoS6RP (Ser235/236) | Cell Signaling Tech-2211 | Polyclonal Ab | WB |  |
| 4EB-P1 | Cell Signaling Tech-9644 | 53H11 | WB |  |
| Phospho4EBP1 (Ser65) | Cell Signaling Tech-9451 | Polyclonal Ab | WB |  |
| PhosphoDrp1 (Ser637) | Cell Signaling Tech-4867 | Polyclonal Ab | IF |  |
| PhosphoDrp1 (Ser616) | Cell Signaling Tech-4494 | Monoclonal Ab | IF |  |
| AMPK | Cell Signaling Tech-2532 | Polyclonal Ab | WB |  |
| Drp1 | Novus 110-55288 | Rabbit polyclonal | WB |  |
| PhosphoAMPK (Thr172) | Cell Signaling Tech-2535 | 40H9 | WB |  |
| ETFDH | Abcam-131376 | 3D1AC4AF3 | WB |  |
| GBE1 | Abcam-180596 | EP11113 | WB |  |
| ACOT2 | NovusBio-NBp1-70402 | Polyclonal Ab | WB |  |
| SLC2A3 | Abcam-191071 | 10508N | WB |  |
| XBP-1s | Biolegend-647501 | Mouse monoclonal | WB |  |
| eiF2Alpha | Enzo ADI-KAP-CP130 | Polyclonal Ab | WB |  |
| phosphoeiF2Alpha | Cell Signaling Tech-9721 | Polyclonal Ab | WB |  |
| CHOP | ThermoFischer-MA1-250 | 9C8 Mouse monoclonal | WB |  |
| ATF4 | Proteintech 10835-1-AP | Polyclonal Ab | WB |  |
| GUF1 | Santa Cruz sc-514604 | Mouse monoclonal | WB |  |
| PDSS2(DLP1) | Santa cruz-515137 | Mouse monoclonal | WB |  |
| ERp72 | Cell Signaling Tech-5033 | D70D12 | IF |  |
| ICSU | ProteinTech 14812-1-AP | Polyclonal Ab | WB |  |
| Hsp60 | Cell Signaling Tech-4869 | Polyclonal ab | WB |  |
| Galectin-3 | Dr H. Leffler | Rat monoclonal | Immuno EM/IF |  |
| Galectin-3 | Abcam 31707 | Polyclonal rabbit | WB |  |
| Tom20 | Santa Cruz Biotech sc 17764 | Mouse monoclonal | WB |  |
| **Antibody anti** | **Provider** | **Clone/source** | **Application** |  |
| SEL1L | Abcam 78298 | Rabbit polyclonal | WB |  |
| FACL4 | Abcam 155282 | Rabbit monoclonal | WB |  |
| β-actin | Sigma AC-15 | Mouse monoclonal | WB |  |
| ATF6 | Abcam 122877 | Mouse monoclonal | WB |  |
|  |  |  |  |  |

**qPCR**

| **Target** | **NM** | **Gene Name** | **TaqMan Gene expression Assay Id** | **Sequence (5’→3’) (F: forward ; R:reverse; P:probe)** |
| --- | --- | --- | --- | --- |
| STC2 | NM_003714 | Stanniocalcin 2 |  | F : GCAGAATACAGCGGAGATCC  R : CAAATCCCATGTAAGCCCCG  P : FAM-GGTGTGGCGTGTTTGAATGT-Tamra |
| XPOT | NM_007235 | Exportin |  | F : CGGGAAGAAGCATGTGACTG  R : TGGCCAGGAAGTCAACATCT  P : FAM-TGGGTTTTTCAGCATTGACCA-Tamra |
| DERL1 | NM_024295 | Derlin 1 |  | F : AGGCCTGCTATTTACCCTGG  R : ACTCCTCCTCTCCTACTGGG  P : FAM-TCAGTTTTTGTACCGCTGGC-Tamra |
| PARN | NM_002582 | poly(A)-specific ribonuclease |  | F : CCTGTCACGATTCCTGAGGA  R : GAACCCGGTACATGGCTCTA  P : FAM-AGTGAAGAAAACAAGAACTTGGA-Tamra |
| USP14 | NM_005151 | Ubiquitin specific peptidase 14 |  | F GCAGTACTAACACACCAGGG  R : CACCACCAGAAAGCCGTAAG  P : FAM-AGTCAGCATCGTAACACCAGA-Tamra |
| TMEM33 | NM_018126 | transmembrane protein 33 |  | F : GGCTTTCTCGCTTGTTCACA  R : TGGCAGCTGTCCTCTAACAA  P : FAM-CTTACCAGTGCTCTGAGGCT-Tamra |
| GFPT1 | NM_002056 | glutamine--fructose-6-phosphate transaminase 1 |  | F : AGCCCTCTGTTGATTGGTGT  R : CACACGAGAGAGATTGCAGC  P : FAM-ACAGAACAGGCAAAGACAAGA-Tamra |
| ETFDH | NM_004453.3 | Electron transfer flavoprotein dehydrogenase | Hs01031780_m1 |  |
| GBE1 | NM_000158.3 | Glucan 1,4 alpha branching enzyme 1 | Hs00609186_m1 |  |
| ACOT2 | NM_006821.5 | Acyl-coA thioesterase 2 | Hs00559940_m1 |  |
| SLC2A3 | NM_006931.2 | Soluble carrier family 2 member 3 | Hs00359840_m1 |  |
| GAPDH | NM_002046.5 | glyceraldehyde-3-phosphate dehydrogenase | Hs99999905_m1 |  |
