# Supplementary figures and images for: Galectin-3 modulates epithelial cell adaptation to stress at the ER-mitochondria interface
Source: Cell Death Dis. 2020 May 12;11(5):360. doi: 10.1038/s41419-020-2556-3 (PMC7217954; doi:10.1038/s41419-020-2556-3)

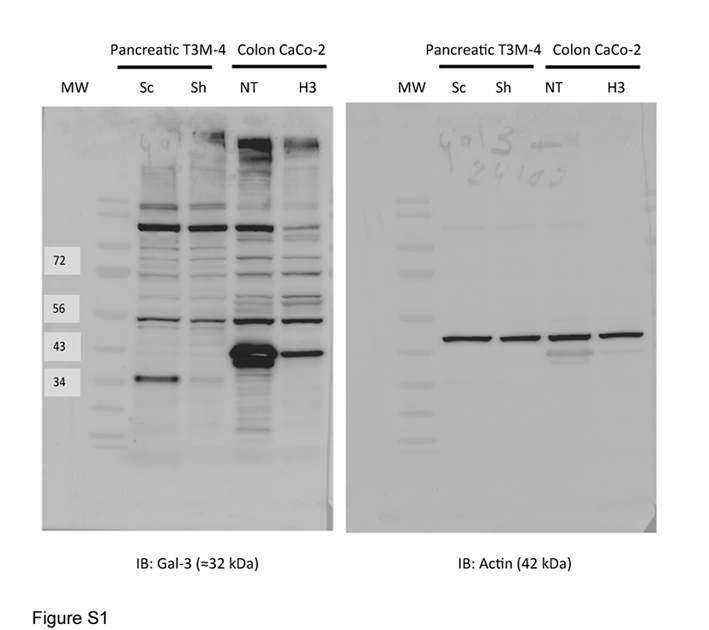

Supplement: Supplementary file 6 — Figure S1 [file 41419_2020_2556_MOESM6_ESM.tif]

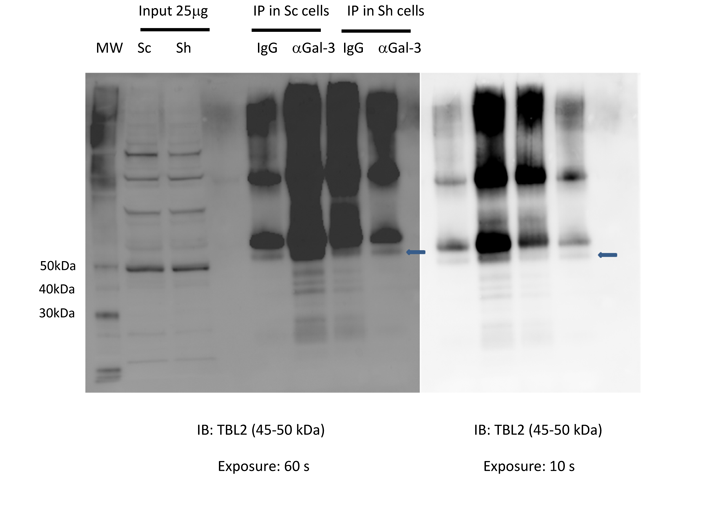

Supplement: Supplementary file 7 — Figure S2 [file 41419_2020_2556_MOESM7_ESM.tif]

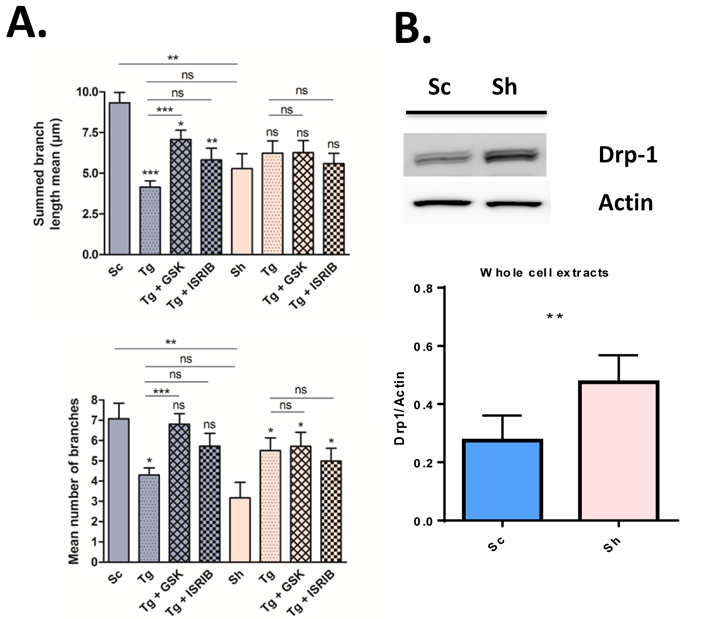

Supplement: Supplementary file 8 — Figure S3 [file 41419_2020_2556_MOESM8_ESM.tif]

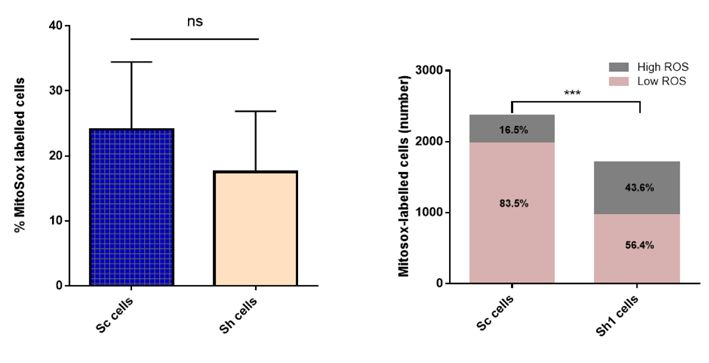

Supplement: Supplementary file 9 — Figure S4 [file 41419_2020_2556_MOESM9_ESM.tif]

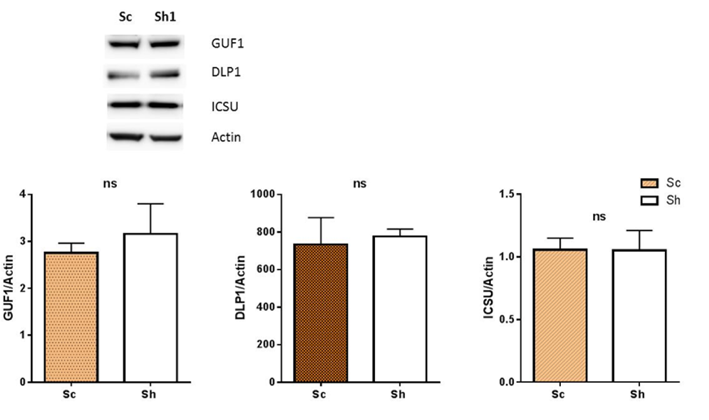

Supplement: Supplementary file 10 — Figure S5 [file 41419_2020_2556_MOESM10_ESM.tif]

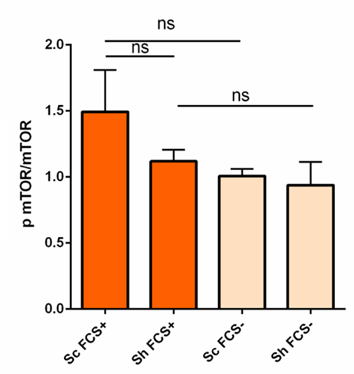

Supplement: Supplementary file 11 — Figure S6 [file 41419_2020_2556_MOESM11_ESM.tif]

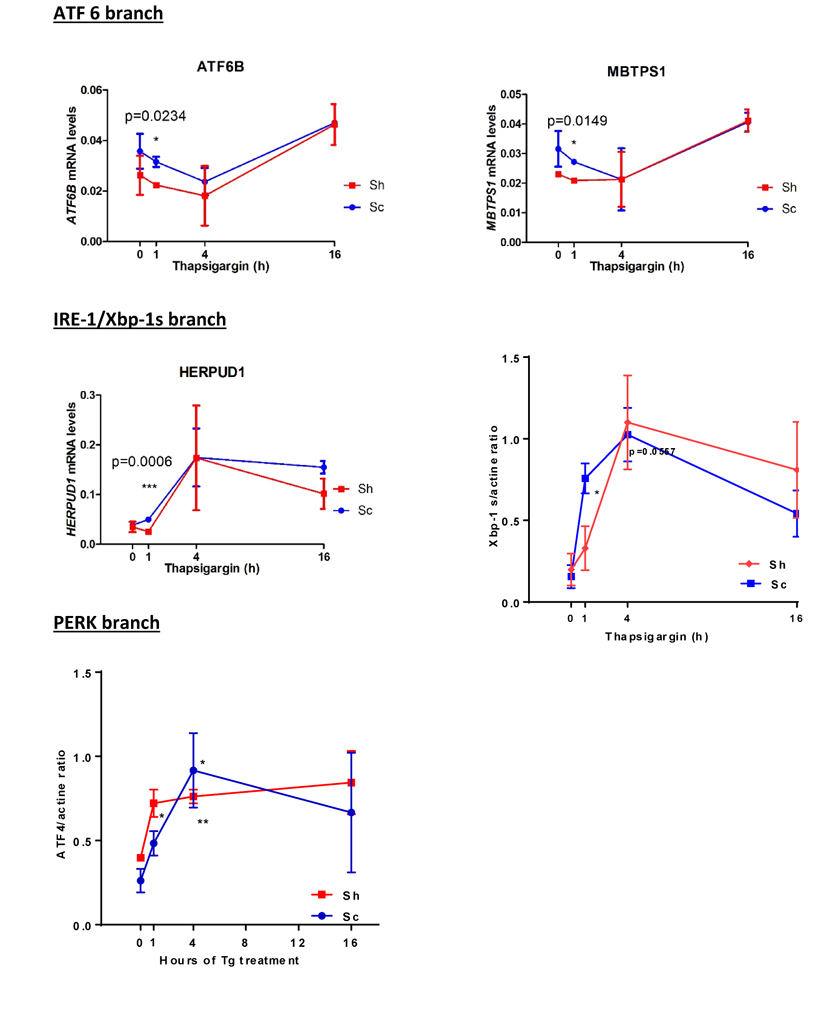

Supplement: Supplementary file 12 — Figure S7 [file 41419_2020_2556_MOESM12_ESM.tif]

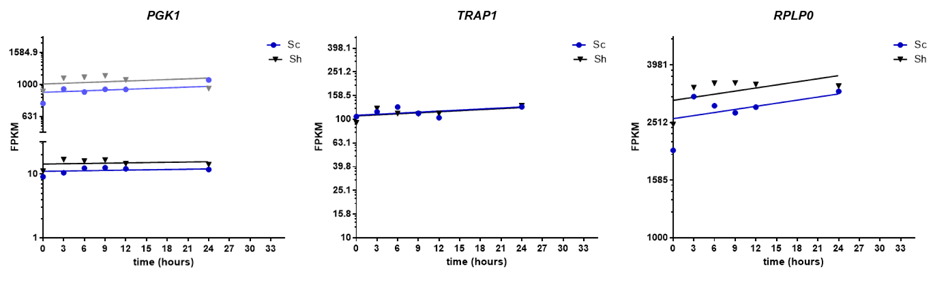

Supplement: Supplementary file 13 — Figure S8 [file 41419_2020_2556_MOESM13_ESM.tif]
